# Sociodemographic Factors and Utilization of Pediatric Oncology Satellite Clinics in Ontario, Canada

**DOI:** 10.1001/jamanetworkopen.2024.52063

**Published:** 2024-12-26

**Authors:** Maria Chiu, Abbas Ali, Felicia Leung, Chaoran Dong, Petros Pechlivanoglou, David Hodgson, Paul Gibson

**Affiliations:** 1 Pediatric Oncology Group of Ontario, Toronto, Ontario, Canada; 2 Institute of Health Policy, Management and Evaluation, University of Toronto, Toronto, Ontario, Canada; 3 Child Health Evaluative Sciences, The Hospital for Sick Children, Peter Gilgan Centre for Research and Learning, Toronto, Ontario, Canada; 4 Department of Radiation Oncology, Princess Margaret Cancer Centre–University Health Network, Toronto, Ontario, Canada; 5 Department of Radiation Oncology, University of Toronto, Toronto, Ontario, Canada; 6 Division of Haematology/Oncology, McMaster Children’s Hospital, Hamilton Health Sciences Centre, Hamilton, Ontario, Canada; 7 Pediatrics, Faculty of Health Sciences, McMaster University, Hamilton, Ontario, Canada

## Abstract

**Importance:**

Pediatric cancer care services in high-income nations are mainly centralized in metropolitan cities. To allow treatments closer to home, patients across Ontario, Canada, a geographically large province, are offered decentralized care via satellite clinics; however, it is unclear whether the utilization of these pediatric oncology satellite clinics differs by area-level sociodemographic factors.

**Objective:**

To examine whether sociodemographic factors, such as area-level income and rurality, are independently associated with the odds of satellite clinic visit and the hazards of time to first visit among pediatric oncology patients receiving cancer treatment.

**Design, Setting, and Participants:**

This is a retrospective population-based cohort study of patients aged 0 to 17 years with cancer living in a satellite catchment area in Ontario, Canada (from 2015 to 2022, with follow-up through 2023). The Pediatric Oncology Group of Ontario Networked Information System cancer registry (containing age, sex, diagnosis type, and year information) was linked to satellite, OpenStreetMap, and postal-code databases to ascertain rural or urban dwelling, neighborhood-based income, and driving time saved.

**Exposures:**

Age, sex, rural or urban dwelling, and neighborhood-level income quintiles.

**Main Outcomes and Measures:**

The primary outcomes were the odds of satellite clinic attendance within 1 year of diagnosis and the hazards of first clinic visit after starting systemic therapy, derived from multivariable logistic and Cox proportional hazards models, respectively.

**Results:**

Among the 1280 eligible patients (median [IQR] age, 7.0 [3.0-13.0] years; 753 male [58.8%]), 844 (65.9%) visited a satellite clinic within 1 year of diagnosis with a median (IQR) of 39 (14-67) days to first visit. Driving time saved (>60 minutes) was the factor most associated with satellite use, followed by diagnostic type (with patients with central nervous system tumors least likely to visit). Rural (vs urban) patients had significantly lower odds of satellite visit within a year of diagnosis (odds ratio, 0.48; 95% CI, 0.31-0.74; *P* = .001) and lower instantaneous likelihood of visiting after start of treatment (hazard ratio, 0.65; 95% CI, 0.53-0.81; *P* < .001). Living in a lower-income (vs middle-income) area was also associated with significantly lower utilization (odds ratio, 0.53; 95% CI, 0.35-0.80; *P* = .009; hazard ratio, 0.73; 95% CI, 0.60-0.89; *P* = .002).

**Conclusions and Relevance:**

This population-based study of pediatric oncology patients found that satellite clinics, despite being designed to reduce transportation and financial burdens, were disproportionately underutilized by patients living in rural and lower-income areas. Monitoring area-level social determinants of health can help inform interventions to improve timely and equitable access to childhood cancer care closer to home.

## Key Points

**Question:**

Does satellite clinic utilization for pediatric cancer care differ by sociodemographic factors?

**Findings:**

In this population-based cohort study of 1280 children with new cancer diagnoses eligible for care at a satellite clinic, rural patients had lower odds of visiting a satellite clinic and lower hazards of first visit vs urban patients. Those living in lower-income areas also disproportionately underutilized satellite clinics vs those in middle-income areas.

**Meaning:**

Despite being designed to reduce transportation and financial burdens, satellite clinics were found to be underutilized by patients living in rural and lower-income areas; these findings may help inform interventions to improve timely and equitable access to cancer care.

## Teaser

This cohort study examines whether, among pediatric oncology patients in Ontario, Canada, area-level income and rurality are factors independently associated with the odds of satellite clinic visit and the hazards of time to first visit.

## Introduction

Each year, approximately 400 children receive a diagnosis of cancer in specialized pediatric cancer centers in Ontario, Canada.^1,2^ Ontario has a population of over 14.2 million and a total land area of more than 1 million square kilometers (approximately 415 000 square miles).^3,4^ Despite Ontario’s publicly funded health care system, its geography presents challenges; approximately 17.2% of the population live outside of metropolitan centers, where Ontario’s specialized pediatric cancer centers are located.^5,6^ Given the province’s vast geography, it is unclear whether pediatric patients with cancer and families living in rural and lower-income areas encounter challenges in accessing cancer treatment and supportive care.

There are currently 5 specialized pediatric cancer centers in Ontario within tertiary hospitals, where the majority of cancer diagnoses and a large proportion of treatment occurs. Geographic location, socioeconomic status, and other social determinants of health can collectively contribute to the complex interplay influencing cancer health services access and outcomes.^7,8,9,10,11^ Studies have demonstrated that greater travel distance from place of residence to cancer care facility can adversely affect patients with cancer, such as stage of diagnosis, treatment modalities used, patient prognosis, financial burden, and overall quality of life.^12,13,14,15,16,17^ Findings from these studies underscore the importance of travel burden, encompassing both distance and time, as crucial factors influencing access to oncology services.^12,13,16,18^ In contrast, a decentralized model can present patients with an opportunity to be managed in a care center with reduced burden and increased service uptake.^18,19,20^

The Pediatric Oncology Group of Ontario (POGO) Satellite Clinic Program is a formal system of 8 satellite clinics that partner with centralized specialized pediatric cancer centers to provide aspects of patients’ cancer care, such as chemotherapy and supportive care, at a hospital closer to home.^21^ On average, satellite patients have a total of 6260 ambulatory visits to satellite clinics and 270 inpatient discharges from satellite hospitals per year. For families, care closer to home means less travel, fewer expenses, and less disruption in daily life, as well as fewer missed school and work days for the family. However, the potential sociodemographic disparities in utilization of these satellite clinics are not known. The objectives of this study were to examine whether sociodemographic factors—namely, age, sex, rurality, and income—were independent factors in the (1) odds of visiting a satellite clinic within a year of diagnosis and (2) hazards of visiting a satellite clinic after initial systemic treatment.

## Methods

### Study Design and Data Sources

We conducted a population-based retrospective cohort study using linked cancer registry, geographic, and satellite clinic databases at POGO.^22^ POGO is a nonprofit organization whose legal status under Ontario’s health information privacy law allows it to collect and analyze health care and demographic data, without consent, for childhood cancer care evaluation and improvement.^22^ Patients were identified from the Pediatric Oncology Group of Ontario Networked Information System (POGONIS), an active population-based registry of children and youth diagnosed with cancer and/or treated in a specialized pediatric cancer center in Ontario.^23^ POGO is a prescribed entity under Ontario’s Personal Health Information Protection Act, Section 45, and as such is authorized to use and disclose deidentified data for health system planning and does not require ethics board review or informed consent. This report followed the Strengthening the Reporting of Observational Studies in Epidemiology (STROBE) reporting guidelines for cohort studies.

Ontario has 8 childhood cancer satellite clinics located in community hospitals. Their catchment areas (eFigure 1 in Supplement 1) were developed on the basis of clinical input regarding referral patterns and drive time from patient residence to the closest childhood cancer program and satellite clinics throughout Ontario using network analysis in ArcGIS.^24^

For this study, we included patients who resided within a satellite catchment area, were aged 0 to 17 years at the time of primary cancer diagnosis between April 1, 2015, and March 31, 2022, and were undergoing a chemotherapy treatment plan. Patients were excluded if they had acute myeloid leukemia (given the intensive, inpatient nature of their treatment regimen), were treated with surgery and/or radiation therapy only (as these services are almost exclusively performed at specialized pediatric cancer centers), or were managed with observation only without active treatment.

### Variable Descriptions

Age at diagnosis, sex at birth, diagnosis type, and year of diagnosis were ascertained from the POGONIS database. Linkage of this cancer registry database to Statistics Canada’s 2017 postal-code conversion file enabled us to ascertain neighborhood-level income quintiles based on before-tax income and urban vs rural dwelling for each patient.^25,26,27,28^ An urban area was defined as one with a population of at least 1000 people and a density of 400 or more people per square kilometer; areas outside urban centers were defined as rural areas.^29^ Diagnoses were grouped into 3 categories from the *International Classification of Childhood Cancer, Third Edition*,^30^ which is based on the *International Classification of Diseases for Oncology* from the International Agency for Research on Cancer: (1) leukemia and lymphoma, (2) solid non–central nervous system (CNS) tumors, and (3) CNS and germ-cell CNS tumors (eTable in Supplement 1). Open Source Routing Machine Application Programming Interface (OSRM API) using OpenStreetMap data was used to determine the quickest route from each postal code to the 8 satellite clinics and 5 specialized pediatric cancer centers, from which we derived the drive time or distance saved, calculated as the shortest drive time or distance to the satellite clinic minus the shortest drive time or distance to the specialized pediatric cancer center.

### Statistical Analysis

Descriptive statistics were used to compare sociodemographic and clinical characteristics between patients who did and did not visit a satellite clinic within 1 year of cancer diagnosis. Percentages and *P* values from χ^2^ tests were reported for categorical variables, and medians, IQRs, and *P* values from Kruskal-Wallis tests were calculated for continuous variables.

To examine access to satellite clinics, we calculated satellite program utilization (the percentage of eligible patients who attended a satellite clinic within 1 year of diagnosis) for each sociodemographic and clinical group, and univariate logistic regression was used to test for significance. For our main analysis, age, sex, rurality, drive time saved, cancer diagnosis category, diagnosis year, and income quintiles were included in a multivariable logistic regression model to estimate odds ratios (ORs) of the association of each factor with satellite clinic attendance within 1 year of cancer diagnosis. In separate sensitivity analyses, we replaced drive time saved with driving distance traveled saved in the logistic regression model and adjusted for the specialized pediatric cancer center (largest vs others).

To assess how quickly a patient sought care at a satellite clinic, we calculated the median (IQR) number of days from the initiation of systemic treatment to first satellite clinic visit for each sociodemographic and clinical group, and the Kruskal-Wallis test was used to test for significance. We then conducted multivariable Cox proportional hazards regression analysis with the aforementioned covariates and sensitivity analyses and time to first satellite visit as the outcome. The proportional hazards assumption was not violated according to an assessment of the log of the negative log of estimated survivor functions and time-dependent variables (ie, interaction between explanatory variables to time).

All statistical analyses were conducted using SAS statistical software version 9.4 (SAS Institute). The level of statistical significance was set to 2-tailed *P* < .05.

## Results

In total, there were 2332 Ontario patients aged 0 to 17 years who received a diagnosis of a primary cancer at a specialized pediatric cancer center between April 1, 2015, and March 31, 2022, and who were undergoing a chemotherapy treatment plan (excluding 68 patients with acute myeloid leukemia and 10 patients with missing values for geographic variables). The patients in this overall population had a median (IQR) age of 6 (2-13) years, 1365 (58.5%) were male, and 1245 (53.4%) had leukemia or lymphoma.

During that period, a total of 1280 patients (55%) lived within 1 of the 8 satellite catchment areas (eFigure 1 in Supplement 1). Those who resided in a catchment area had a median (IQR) age of 7.0 (3.0-13.0) years, 753 (58.8%) were male, and 694 (54.2%) had diagnoses of leukemia or lymphoma. Among those who resided in a satellite catchment area, 844 patients (65.9%) had at least 1 visit to a satellite clinic within 1 year of diagnosis; the median (IQR) time saved was 24.9 (17.4-55.8) minutes, and the median (IQR) distance saved was 29.8 (19.4-64.9) km (Table 1). The median (IQR) follow-up time for patients who visited satellite clinics was 1.3 (0.5-2.2) months, and the median (IQR) follow-up time for those who did not visit was 3.4 (1.9-5.7) years.

**Table 1.** Characteristics of Children and Youth in Ontario, Canada, With a First Primary Cancer Diagnosed Between April 1, 2015, and March 31, 2022, Who Resided in Satellite Catchment Areas

Characteristics	Patients, No. (%)			*P* value
	Total (N = 1280 ([100.0%])	Visited a satellite clinic within 1 y of diagnosis (n = 844 [65.9%])	Did not visit a satellite clinic within 1 y of diagnosis (n = 436 [34.1%])	
Age at diagnosis, y				
Median (IQR)	7.0 (3.0-13.0)	7.0 (3.0-13.0)	6.0 (2.0-13.0)	.27
0-4	498 (38.9)	329 (39.0)	169 (38.8)	.71
5-9	262 (20.5)	166 (19.7)	96 (22.0)	
10-14	311 (24.3)	206 (24.4)	105 (24.1)	
15-17	209 (16.3)	143 (16.9)	66 (15.1)	
Sex				
Female	527 (41.2)	357 (42.3)	170 (39.0)	.25
Male	753 (58.8)	487 (57.7)	266 (61.0)	
Diagnosis group				
Leukemia and lymphoma	694 (54.2)	461 (54.6)	233 (53.4)	<.001
Solid (non-CNS) tumors	374 (29.2)	273 (32.3)	101 (23.2)	
CNS and germ cell CNS tumors	212 (16.6)	110 (13.0)	102 (23.4)	
Residential location				
Urban	1150 (89.8)	761 (90.2)	389 (89.2)	.60
Rural	130 (10.2)	83 (9.8)	47 (10.8)	
Drive time or distance saved^a^				
Time from residence to satellite clinic, median (IQR), min	24.1 (13.6-34.9)	23.7 (12.2-35.0)	25.3 (16.0-34.7)	.003
Distance from residence to satellite clinic, median (IQR), km	22.3 (10.6-36.7)	21.4 (9.0-36.4)	23.7 (13.2-36.9)	.003
Time saved from residence to satellite clinic vs specialized childhood cancer center, min				
Median (IQR)	22.0 (16.0-46.3)	24.9 (17.4-55.8)	17.4 (9.7-27.5)	<.001
<0	22 (1.7)	6 (0.7)	16 (3.7)	<.001
0-30	789 (61.6)	469 (55.6)	320 (73.4)	
31-60	244 (19.1)	183 (21.7)	61 (14.0)	
61-120	98 (7.7)	80 (9.5)	18 (4.1)	
>120	127 (9.9)	106 (12.6)	21 (4.8)	
Distance saved from residence to satellite clinic vs specialized childhood cancer center, km				
Median (IQR)	25.1 (17.2-55.0)	29.8 (19.4-64.9)	20.3 (19.4-32.3)	<.001
<0	57 (4.5)	25 (3.0)	32 (7.3)	<.001
0-50	867 (67.7)	530 (62.8)	337 (77.3)	
51-100	160 (12.5)	128 (15.2)	32 (7.3)	
101-150	62 (4.8)	50 (5.9)	12 (2.8)	
>150	134 (10.5)	111 (13.2)	23 (5.3)	
Neighborhood income quintile^b^				
Quintile 1 (lowest)	166 (13.0)	97 (11.5)	69 (15.8)	.21
Quintile 2	221 (17.3)	146 (17.3)	75 (17.2)	
Quintile 3	305 (23.8)	212 (25.1)	93 (21.3)	
Quintile 4	312 (24.4)	207 (24.5)	105 (24.1)	
Quintile 5 (highest)	276 (21.6)	182 (21.6)	94 (21.6)	

Abbreviation: CNS, central nervous system.

^a^ Drive time or distance saved is calculated as the shortest drive time or distance to the satellite clinic minus the shortest drive time or distance to specialized pediatric cancer center. Patients whose drive time to a specialized pediatric cancer center is shorter than their drive time to a satellite clinic will have drive time saved values less than 0 minutes.

^b^ These quintiles are defined according to the distribution of before-tax income within each census metropolitan area, census agglomeration, or residual area within Ontario based on 2016 Census (Statistics Canada) data.^28^

The satellite program utilization ranged from 51.9% (95% CI, 45.2%-58.6%) for patients with CNS and germ-cell CNS tumors to 73.0% (95% CI, 68.5%-77.5%) for those with non-CNS solid tumors across diagnostic groups, and from 59.4% (95% CI, 56.0-62.9) for 0 to 30 minutes to 83.5% (95% CI, 77.0%-89.9%) for more than 120 minutes across categories of drive time saved (Table 2). The lowest satellite program utilization was observed among patients in the lowest-income quintile areas (58.4%; 95% CI, 50.9%-65.9%) and the highest among those in the middle-income areas, quintile 3 (69.5%; 95% CI, 64.3%-74.7%); the latter was used as the reference for multivariable analyses (Table 2).

**Table 2.** Satellite Program Utilization, Within the First Year of Diagnosis, Among Children and Youth With a First, Primary Cancer Diagnosed Between April 1, 2015, and March 31, 2022, Who Resided in Satellite Catchment Areas

Characteristics	Patients, No.	Satellite program utilization, % (95% CI)	*P* value
Total population	1280	65.9 (63.3-68.5)	Not applicable
Age at diagnosis, y			
0-4	498	66.1 (61.9-70.2)	Reference
5-9	262	63.4 (57.5-69.2)	.65
10-14	311	66.2 (61.0-71.5)	.93
15-17	209	68.4 (62.1-74.7)	.57
Sex			
Female	527	67.7 (63.8-71.7)	Reference
Male	753	64.7 (61.3-68.1)	.25
Diagnosis group			
Leukemia and lymphoma	694	66.4 (62.9-69.9)	Reference
Solid (non-CNS) tumors	374	73.0 (68.5-77.5)	<.001
CNS and germ cell CNS tumors	212	51.9 (45.2-58.6)	<.001
Residential location			
Urban	1150	66.2 (63.4-68.9)	Reference
Rural	130	63.8 (55.6-72.1)	.60
Drive time and distance saved^a^			
Time saved from residence to satellite clinic vs specialized pediatric cancer center, min			
<0	22	27.3 (8.7-45.9)	<.001
0-30	789	59.4 (56.0-62.9)	Reference
31-60	244	75.0 (69.6-80.4)	.02
61-120	98	81.6 (74.0-89.3)	<.001
>120	127	83.5 (77.0-89.9)	<.001
Distance saved from residence to satellite clinic vs specialized pediatric cancer center, km			
<0	57	43.9 (31.0-56.7)	<.001
0-50	867	61.1 (57.9-64.4)	Reference
51-100	160	80.0 (73.8-86.2)	.01
101-150	62	80.6 (70.8-90.5)	.06
>150	134	82.8 (76.5-89.2)	.001
Neighborhood income quintile			
Quintile 1 (lowest)	166	58.4 (50.9-65.9)	.03
Quintile 2	221	66.1 (59.8-72.3)	.80
Quintile 3	305	69.5 (64.3-74.7)	Reference
Quintile 4	312	66.3 (61.1-71.6)	.69
Quintile 5 (highest)	276	65.9 (60.4-71.5)	.82
Diagnosis period			
April 1, 2015, to March 31, 2016	210	62.9 (56.3-69.4)	Reference
April 1, 2016, to March 31, 2017	166	68.7 (61.6-75.7)	.45
April 1, 2017, to March 31, 2018	175	69.1 (62.3-76.0)	.36
April 1, 2018, to March 31, 2019	189	69.3 (62.7-75.9)	.31
April 1, 2019, to March 31, 2020	181	68.0 (61.2-74.8)	.57
April 1, 2020, to March 31, 2021	177	63.8 (56.8-70.9)	.49
April 1, 2021, to March 31, 2022	182	60.4 (53.3-67.5)	.08

Abbreviation: CNS, central nervous system.

^a^ Drive time or distance saved is calculated as the shortest drive time or distance to the satellite clinic minus the shortest drive time or distance to specialized pediatric cancer center. Patients whose drive time to a specialized pediatric cancer center is shorter than their drive time to a satellite clinic will have drive time saved values less than 0 minutes.

**Figure 1.**
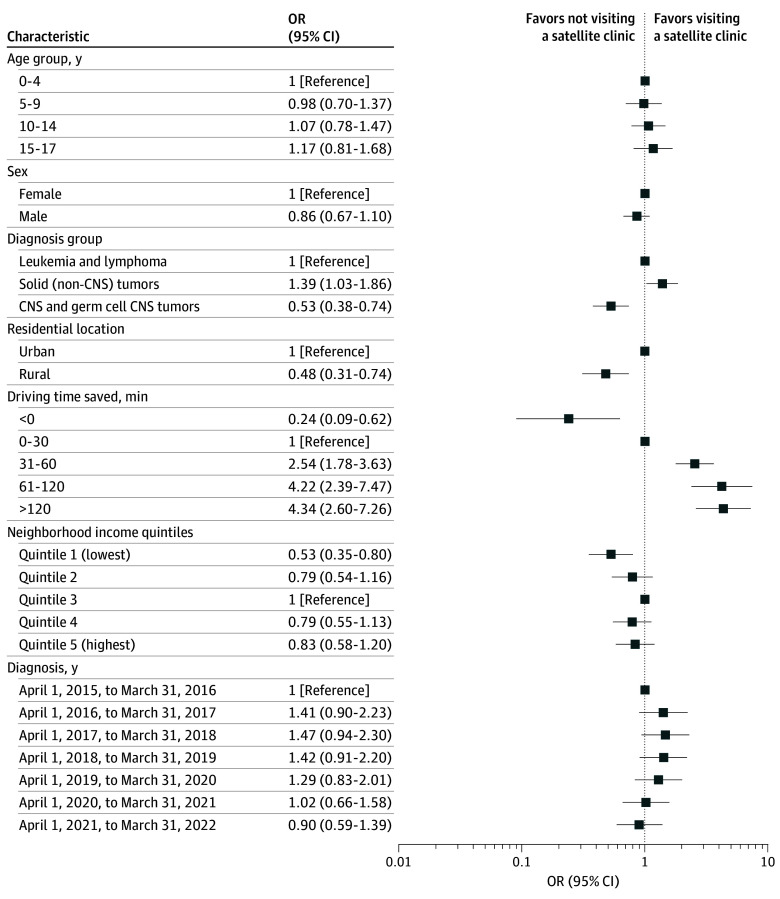
Odds Ratios (ORs) for Likelihood of Visiting a Satellite Clinic Within 1 Year of Diagnosis ORs are adjusted for geographic (time saved), sociodemographic, and clinical factors, among 1280 children with a first primary cancer diagnosed between April 1, 2015, and March 31, 2022, in Ontario, Canada. CNS indicates central nervous system.

When covariates were added to a multivariable logistic regression model, significantly lower odds of satellite visit within 1 year of diagnosis were observed among patients with CNS and germ-cell CNS tumors compared with patients with leukemia or lymphoma (OR, 0.53; 95% CI, 0.38-0.74; *P* < .001), among rural patients compared with urban dwellers (OR, 0.48; 95% CI, 0.31-0.74; *P* = .001), and among those living in the lowest-income quintile compared with middle-income quintile neighborhoods (OR, 0.53; 95% CI, 0.35-0.80; *P* = .009) (Figure 1). Patients with non-CNS solid tumors were more likely to visit a satellite clinic within 1 year after diagnosis compared with patients with leukemia or lymphoma (OR, 1.39; 95% CI, 1.03-1.86; *P* < .001). Patients who saved more than 30 minutes drive time had a 2.54 (95% CI, 1.78-3.63; *P* = .01) to 4.34 (95% CI, 2.60-7.26; *P* < .001) greater odds of visiting a satellite clinic than patients who saved less than 30 minutes (Figure 1). There was no significant difference in the likelihood of a satellite visit across age, sex, and diagnosis period.

Among the 858 patients who made a satellite visit after their first systemic treatment, the median (IQR) time to first visit was 39 (14-67) days (Table 3). The shortest median (IQR) times to satellite clinic visit were observed among 15- to 17-year-olds (19 [9-45] days), urban patients (39 [14-64] days), and patients with solid tumors (13 [7-31] days) (Table 3). The proportion of patients who visited a satellite clinic at 1 and 2 years were 58.9% (95% CI, 50.1%-65.0%) and 69.3% (95% CI, 60.4%-77.8%) for rural patients and 62.6% (95% CI, 59.7%-65.5%) and 70.4% (95% CI, 67.5%-73.2%) for urban patients. For area-based income, the proportion who visited at 1 and 2 years were 51.1% (95% CI, 43.3%-59.5%) and 59.9% (95% CI, 51.9%-68.1%) for the lowest-income quintile, 67.5% (95% CI, 62.0%-72.9%) and 76.3% (95% CI, 71.0%-81.1%) for the middle-income quintile, and 62.3% (95% CI, 56.3%-68.4%) and 70.8% (95% CI, 64.9%-76.5%) for the highest-income quintile.

**Table 3.** Time Between Start of Treatment and First Visit to a Satellite Clinic Among 858 Children and Youth With a First, Primary Cancer Diagnosed Between April 1, 2015, and March 31, 2022, Who Visited a Satellite Clinic During the Study Follow-Up

Characteristics	Time from treatment start to first satellite clinic visit, median (IQR), d	*P* value
Total population	39 (14-67)	Not applicable
Age at diagnosis, y		
0-4	43 (23-73)	<.001
5-9	46 (32-88)	
10-14	37 (11-58)	
15-17	19 (9-45)	
Sex		
Female	39 (13-63)	.22
Male	39 (16-70)	
Diagnosis group		
Leukemia and lymphoma	45 (34-70)	<.001
Solid (non-CNS) tumors	13 (7-31)	
CNS and germ cell CNS tumors	72 (38-141)	
Residential location		
Urban	39 (14-64)	.01
Rural	49 (29-91)	
Distance and driving time		
Time saved from residence to satellite clinic vs specialized pediatric cancer center, min		
<0	34 (27-49)	.95
0-30	40 (15-64)	
31-60	41 (13-73)	
61-120	37 (18-71)	
>120	37 (12-73)	
Distance saved from residence to satellite clinic vs specialized pediatric cancer center, km		
<0	34 (16-53)	.18
0-50	39 (14-63)	
51-100	47 (24-78)	
101-150	36 (17-66)	
>150	36 (12-73)	
Neighborhood income quintile		
Quintile 1 (lowest)	49 (20-90)	.07
Quintile 2	41 (13-60)	
Quintile 3	41 (15-63)	
Quintile 4	36 (13-58)	
Quintile 5 (highest)	39 (13-72)	
Diagnosis period		
April 1, 2015, to March 31, 2016	40 (20-102)	.25
April 1, 2016, to March 31, 2017	43 (18-84)	
April 1, 2017, to March 31, 2018	36 (11-63)	
April 1, 2018 to March 31, 2019	39 (13-65)	
April 1, 2019, to March 31, 2020	40 (16-68)	
April 1, 2020, to March 31, 2021	40 (15-56)	
April 1, 2021, to March 31, 2022	38 (11-58)	

Abbreviation: CNS, central nervous system.

**Figure 2.**
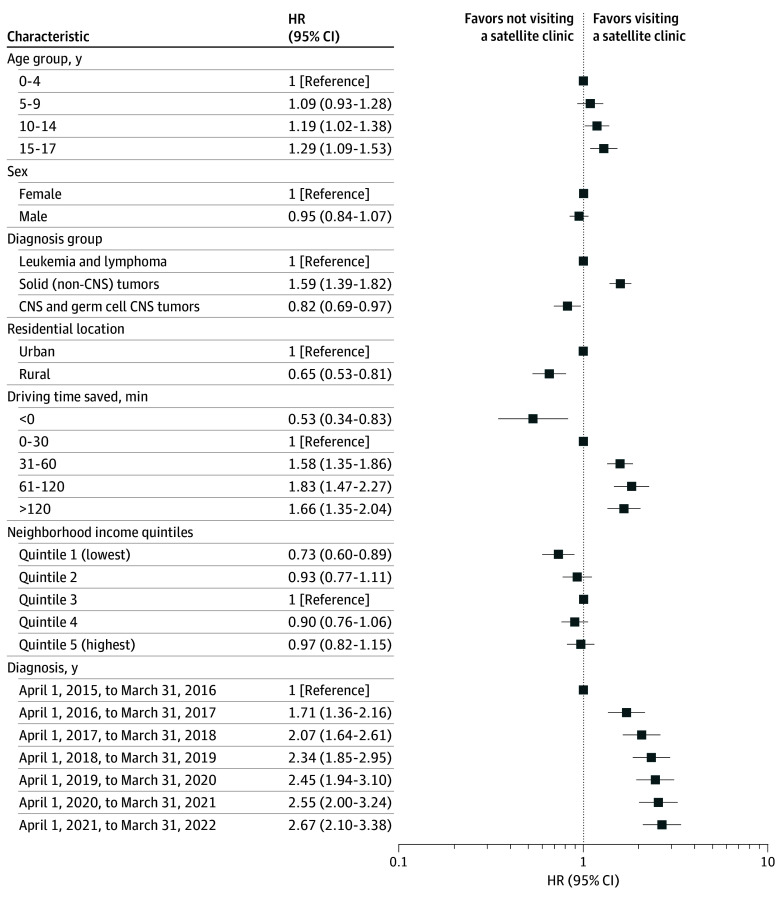
Hazard Ratios (HRs) for Likelihood of Visiting a Satellite Clinic After Starting Systemic Therapy HRs are adjusted for geographic (time saved) and sociodemographic factors among 1253 children and youth with a first primary cancer diagnosed between April 1, 2015, and March 31, 2022, in Ontario, Canada. CNS indicates central nervous system.

After mutually adjusting for all covariates in the multivariable Cox proportional hazards model, older age groups (10-17 years old) and patients with non-CNS solid tumors had significantly greater hazards of satellite clinic visits than younger patients and those with leukemia or lymphoma (Figure 2). Patients living in rural areas (hazard ratio, 0.65; 95% CI, 0.53-0.81; *P* < .001) and the lowest-income areas (hazard ratio, 0.73; 95% CI, 0.60-0.89; *P* = .002) had significantly lower instantaneous likelihood of visiting a satellite clinic after start of treatment than their respective comparison groups. There was a graded association by diagnosis year, where the hazard of first visit improved from 2015 to 2022. Patients who saved more than 30 minutes of drive time had significantly higher hazards of visiting a satellite clinic compared with those who saved less than 30 minutes. No significant difference by sex was observed (Figure 2). For the sensitivity analyses, similar model estimates emerged when adjusting for distance saved instead of driving time saved (eFigure 2 and eFigure 3 in Supplement 1) or when models were additionally adjusted for specialized pediatric cancer center (rural, OR, 0.54; 95% CI, 0.33-0.86; lowest-income quintile, OR, 0.49; 95% CI, 0.32-0.77).

## Discussion

Most pediatric cancer care services in high-income nations are centralized in metropolitan cities, where advanced treatments and specialists are accessible to a larger population.^31,32,33^ For patients and caregivers who reside in remote and rural areas, this centrality of care creates challenges, including more time and financial resources spent for traveling and lodging.^31,32^ In this population-based cohort study within Ontario’s universal health care system, we found disparities among patients living in rural and lower income areas in utilization of satellite clinics intended to provide childhood cancer care closer to home. Compared with their counterparts, patients living in rural and the lowest income areas had significantly lower odds and hazards of visiting a satellite clinic. A diagnosis of a non-CNS solid tumor and greater than 30 minutes drive time saved were significantly associated with greater odds and hazards of satellite clinic utilization. There was no significant difference by sex in satellite clinic use.

The POGO satellite program was designed to provide care to patients closer to home by reducing the need to travel to specialized pediatric cancer centers. To our knowledge, there is a dearth of information in the literature about decentralization of childhood cancer care. Evidence from the adult cancer literature shows that US patients living far from tertiary hospitals face financial burdens related to transportation and accommodation costs and challenges in taking time off work and feeling isolated from their loved ones.^34^ US patients using chemotherapy infusion sites at local centers^34^ and South African patients attending oncology clinics closer to home^35^ reported fewer barriers to care and better quality of life. The decentralized model has also been shown to be effective in reducing barriers to access to care for adult kidney dialysis,^36^ in vitro fertilization,^37^ and HIV treatment.^38^ Providing treatment closer to home is particularly important for children, youth, and their families, minimizing disruption to their daily lives as they navigate managing their disease. Closer access to care means children can be closer to the comforts of home, their families, schools, and support system and can help reduce burdens on caregivers who are already balancing multiple responsibilities. Furthermore, this allows resources and hospital beds to be freed up at tertiary hospitals. The satellite model was designed to provide an opportunity to access care without extensive travel and to minimize school days missed by patients and missed workdays by parents and families; therefore, it is important that our study identified prevailing disparities by geography and neighborhood income even after accounting for differences in age, diagnosis, and travel time saved.

We found that rurality was an independent factor associated with lower odds of and delays in visits to decentralized satellite clinics. Some rural patients face considerable travel distances to either tertiary hospitals or satellite clinics, with some needing to travel by airplane to reach a treatment facility. Given the substantial travel distance and time to reach either a tertiary or satellite facility, patients may opt to travel to specialized pediatric centers in tertiary hospitals, where arranging travel and accommodation near metropolitan areas may be less challenging. This preference may also arise from the perception that tertiary centers, being academic institutions in larger cities, offer better quality and more comprehensive care.

Income is a key social determinant of health because it impacts access to other social determinants of health, including housing, food security, and health services.^39,40^ Our study showed that, despite Ontario’s universal and publicly funded health care system, children and youth living in the lowest-income quintile areas were least likely to utilize cancer satellite clinics. This finding is comparable to the association seen in adult cancer populations. A recent meta-analysis^41^ of 13 studies from the US and Europe on socioeconomic status and pancreatic cancer found that lower socioeconomic status was significantly associated with reduced access to cancer surgery, chemotherapy, and radiation therapy. An area-based measure of lower income and racial privilege was also shown to be associated with lower access to complex cancer surgery at high-volume centers in the US.^42^ In the US, a lack of insurance coverage among individuals of lower socioeconomic status has been identified as a primary barrier to accessing health care.^41,43^ However, studies from European countries, which have universal health care systems, show similar patterns, suggesting that factors other than insurance coverage may mediate the association of income with access to cancer care.^41^ An interesting finding from our study that warrants additional investigation is a potential threshold association of neighborhood income with access to decentralized childhood cancer care. Patients living in areas with the median neighborhood income quintile were most likely to use satellite clinics compared with the lowest and highest income quintiles, suggesting there are characteristics of families that reach the median neighborhood income that may make them more likely to visit satellite clinics, such as transportation means, educational background, health literacy, and employment conditions, including flexible work hours and paid caregiver days. Future qualitative studies are needed to elucidate reasons for lower utilization among high-income families and barriers that low-income families face in accessing decentralized care. There may also be opportunities to improve the referral process to target lower income families, because the outcomes of not accessing care closer to home are likely compounded in lower income groups, who are already among the most vulnerable members of the education system and workforce.^44,45,46,47^

As expected, we found that satellite clinic utilization was lowest among patients with CNS tumors and highest among patients with non-CNS solid tumors. These findings are consistent with clinical differences in disease type and treatment regimens, with patients with CNS tumors being more likely to have ongoing sequelae from surgery, thus necessitating a closer follow-up at a specialized cancer care center and those with non-CNS solid tumors having multiple chemotherapy cycles that can be provided in satellite clinics. The longer time to first satellite clinic visit observed in the leukemia and lymphoma group also provides face validity to our findings, as patients with acute lymphoblastic leukemia, the most common type of childhood cancer, are not recommended to visit satellite clinics during the induction phase of their treatment when patients are immunosuppressed and at greater risk of infections.

### Limitations

There are some limitations to this study. First, we lacked data on other relevant sociodemographic characteristics, such as race, ethnicity, English language proficiency, family-level income, and educational attainment, which previous studies have found to impact health care access.^39,40,48,49^ Second, our study may not be generalizable to patients undergoing treatment plans that were excluded from the study cohort. However, the relative number of these patients is small, and they are more likely to be followed by a specialized pediatric cancer center. Nonetheless, a strength of this study is our ability to examine access to care indicators by the main types of childhood cancer diagnoses in a population-based cohort derived from a comprehensive cancer registry. Third, our driving duration and distance were computed using OSRM API, ensuring that the optimal route between postal codes and pediatric care facilities was determined; however, OSRM does not account for public transportation modes and does not incorporate real-time dynamic traffic conditions (eg, rush hour). Fourth, we did not have information about the reasons for clinic nonreferral or nonattendance, or patient and family preferences, which are factors that may influence satellite program utilization beyond geographic proximity and financial means.

## Conclusions

Disparities among patients living in rural and lower income areas warrant attention and may inform future satellite site planning and targeted patient outreach. Satellite clinic care represents a departure from traditional centralized health care systems and emphasizes decentralization of childhood cancer care and community-based service provision to enhance accessibility to care. The findings presented here from Ontario, Canada, may be used by other jurisdictions to improve access to pediatric cancer care. Monitoring area-level social determinants of health that influence access to essential treatment and supportive care during the childhood cancer journey can help inform interventions to improve timely and equitable access to cancer care.

## Notes

Supplement 1. **eTable**. International Classification of Childhood Cancer (ICCC) Diagnostic groups **eFigure 1.** The geographic catchment areas around the Pediatric Oncology Group of Ontario’s eight satellite clinics **eFigure 2.** Sensitivity Analysis - Odds ratios for visiting a satellite clinic within one year of diagnosis, adjusted for geographic (distance-saved), socio-demographic and clinical factors, among children and youth diagnosed with a first, primary cancer between April 1, 2015, and March 31, 2022, in Ontario (n=1,280) **eFigure 3.** Sensitivity Analysis - Hazard ratios for visiting a satellite clinic after starting systemic therapy. adjusted for geographic (distance-saved), socio-demographic and clinical factors, among children and youth diagnosed with a first, primary cancer between April 1, 2015, and March 31, 2022, in Ontario (n=1,253)

Supplement 2.
Data Sharing Statement

## References

[1] Centre for Surveillance and Applied Research, Public Health Agency of Canada. Cancer in young people in Canada data tool. 2024. Accessed May 1, 2024. https://health-infobase.canada.ca/data-tools/cypc/

[2] Pediatric Oncology Group of Ontario (POGO). Childhood cancer in Ontario: the 2020 surveillance report. 2020. Accessed May 1, 2024. https://www.pogo.ca/research-data/data-reports/2020-pogo-surveillance-report/

[3] Statistics Canada. Census profile, 2021 census of population. Catalogue No. 98-316-X2021001. Published February 9, 2022. Updated November 15, 2023. Accessed May 1, 2024. https://www12.statcan.gc.ca/census-recensement/2021/dp-pd/prof/index.cfm?Lang=E

[4] Government of Ontario. About Ontario. 2024. Accessed May 1, 2024. https://www.ontario.ca/page/about-ontario

[5] Statistics Canada. Canada’s population estimates: subprovincial areas, July 1, 2023. May 22, 2024. Accessed May 1, 2024. https://www150.statcan.gc.ca/n1/daily-quotidien/240522/dq240522b-eng.htm

[6] Statistics Canada. Population growth in Canada’s rural areas, 2016 to 2021. February 9, 2022. Accessed May 1, 2024. https://www12.statcan.gc.ca/census-recensement/2021/as-sa/98-200-x/2021002/98-200-x2021002-eng.cfm

[7] Tran YH, Coven SL, Park S, Mendonca EA. Social determinants of health and pediatric cancer survival: a systematic review. Pediatr Blood Cancer. 2022;69(5):e29546. doi:10.1002/pbc.29546 35107854 PMC8957569

[8] Onega T, Alford-Teaster J, Wang F. Population-based geographic access to parent and satellite National Cancer Institute Cancer Center Facilities. Cancer. 2017;123(17):3305-3311. doi:10.1002/cncr.30727 28464212

[9] Huang LC, Ma Y, Ngo JV, Rhoads KF. What factors influence minority use of National Cancer Institute-designated cancer centers? Cancer. 2014;120(3):399-407. doi:10.1002/cncr.28413 24452674 PMC3905240

[10] de Souza JA, Hunt B, Asirwa FC, Adebamowo C, Lopes G. Global health equity: cancer care outcome disparities in high-, middle-, and low-income countries. J Clin Oncol. 2016;34(1):6-13. doi:10.1200/JCO.2015.62.2860 26578608 PMC5795715

[11] Morris AM, Rhoads KF, Stain SC, Birkmeyer JD. Understanding racial disparities in cancer treatment and outcomes. J Am Coll Surg. 2010;211(1):105-113. doi:10.1016/j.jamcollsurg.2010.02.051 20610256

[12] Ambroggi M, Biasini C, Del Giovane C, Fornari F, Cavanna L. Distance as a barrier to cancer diagnosis and treatment: review of the literature. Oncologist. 2015;20(12):1378-1385. doi:10.1634/theoncologist.2015-0110 26512045 PMC4679078

[13] Rocque GB, Williams CP, Miller HD, . Impact of travel time on health care costs and resource use by phase of care for older patients with cancer. J Clin Oncol. 2019;37(22):1935-1945. doi:10.1200/JCO.19.00175 31184952 PMC6804875

[14] Kobayashi D, Otsubo T, Imanaka Y. The effect of centralization of health care services on travel time and its equality. Health Policy. 2015;119(3):298-306. doi:10.1016/j.healthpol.2014.11.008 25480458

[15] Lin CC, Bruinooge SS, Kirkwood MK, . Association between geographic access to cancer care, insurance, and receipt of chemotherapy: geographic distribution of oncologists and travel distance. J Clin Oncol. 2015;33(28):3177-3185. doi:10.1200/JCO.2015.61.1558 26304878 PMC4979096

[16] George M, Ngo P, Prawira A. Rural oncology: overcoming the tyranny of distance for improved cancer care. J Oncol Pract. 2014;10(3):e146-e149. doi:10.1200/JOP.2013.001228 24667293

[17] Tarnasky AM, Olivere LA, Ledbetter L, Tracy ET. Examining the effect of travel distance to pediatric cancer centers and rurality on survival and treatment experiences: a systematic review. J Pediatr Hematol Oncol. 2021;43(5):159-171. doi:10.1097/MPH.0000000000002095 33625091

[18] Iverson KR, Svensson E, Sonderman K, . Decentralization and regionalization of surgical care: a review of evidence for the optimal distribution of surgical services in low- and middle-income countries. Int J Health Policy Manag. 2019;8(9):521-537. doi:10.15171/ijhpm.2019.43 31657175 PMC6815989

[19] Nyangasi MF, McLigeyo AA, Kariuki D, Mithe S, Orwa A, Mwenda V. Decentralizing cancer care in sub-Saharan Africa through an integrated regional cancer centre model: the case of Kenya. PLOS Glob Public Health. 2023;3(9):e0002402. doi:10.1371/journal.pgph.0002402 37738236 PMC10516416

[20] Myklebust LH, Olstad R, Bjorbekkmo S, Eisemann M, Wynn R, Sørgaard K. Impact on continuity of care of decentralized versus partly centralized mental health care in Northern Norway. Int J Integr Care. 2011;11:e142. doi:10.5334/ijic.674 22359521 PMC3280921

[21] Pediatric Oncology Group of Ontario (POGO). POGO provincial pediatric oncology satellite manual. 2022. Accessed May 1, 2024. https://www.pogo.ca/satellite-manual/

[22] Pediatric Oncology Group of Ontario (POGO). Accessed November 14, 2024. http://www.pogo.ca

[23] Pediatric Oncology Group of Ontario. Pediatric Oncology Group of Ontario Networked Information System (POGONIS). March 2024. Accessed May 1, 2024. https://www.pogo.ca/research-data/databases/pogonis-childhood-cancer-database/

[24] Environmental Systems Research Institute. ArcGIS Arch Map Desktop: release 11.8.2. 2021. Accessed November 14, 2024. https://desktop.arcgis.com/en/arcmap/latest/get-started/installation-guide/installing-on-your-computer.htm

[25] Statistics Canada. Postal CodeOM Conversion File (PCCF). Statistics Canada Catalogue no. 92-154-X. 2017. Accessed November 14, 2024. https://www150.statcan.gc.ca/n1/en/catalogue/92-154-X

[26] Postal Code Conversion Files (PCCF). MacOdrum Library. Accessed May 1, 2024. https://library.carleton.ca/guides/help/postal-code-conversion-files-pccf

[27] Postal CodeOM Conversion File (PCCF). Reference Guide. Statistics Canada Catalogue no. 92-154-G. December 13, 2017. Accessed May 1, 2024. https://www150.statcan.gc.ca/n1/pub/92-154-g/92-154-g2017001-eng.htm

[28] Canadian Institute for Health Information. Measuring health inequalities: a toolkit. Area-Level Equity Stratifiers Using PCCF and PCCF+. 2018. Accessed November 15, 2024. https://www.cihi.ca/sites/default/files/document/toolkit-area-level-measurement-pccf-en.pdf

[29] Statistics Canada. Population Centre. 2023. Accessed March 28, 2024. https://www12.statcan.gc.ca/census-recensement/2021/ref/dict/az/Definition-eng.cfm?ID=geo049a

[30] Steliarova-Foucher E, Colombet M, Ries L, Rous B, Stiller C. International Incidence of Childhood Cancer, Volume III. International Agency for Research on Cancer. Accessed November 14, 2024. https://iicc.iarc.fr/

[31] Fluchel MN, Kirchhoff AC, Bodson J, . Geography and the burden of care in pediatric cancers. Pediatr Blood Cancer. 2014;61(11):1918-1924. doi:10.1002/pbc.25170 25131518 PMC4749153

[32] Liu X, Fluchel MN, Kirchhoff AC, Zhu H, Onega T. Geographic access to pediatric cancer care in the US. JAMA Netw Open. 2023;6(1):e2251524. doi:10.1001/jamanetworkopen.2022.51524 36656577 PMC9856631

[33] Nguyen CA, Beaulieu ND, Wright AA, Cutler DM, Keating NL, Landrum MB. Organization of cancer specialists in US physician practices and health systems. J Clin Oncol. 2023;41(26):4226-4235. doi:10.1200/JCO.23.00626 37379501 PMC10852402

[34] Curtis ML, Eschiti VS. Geographic health disparities: satellite clinics for cancer care in rural communities. Clin J Oncol Nurs. 2018;22(5):500-506. doi:10.1188/18.CJON.500-506 30239508

[35] Jojo LW, Nkutu NT. Experiences of patients on cancer treatment regarding decentralization of oncology services at a tertiary hospital in the Eastern Cape. BMC Cancer. 2023;23(1):453. doi:10.1186/s12885-023-10876-5 37202732 PMC10195123

[36] Diamant MJ, Young A, Gallo K, . Hemodialysis in a satellite unit: clinical performance target attainment and health-related quality of life. Clin J Am Soc Nephrol. 2011;6(7):1692-1699. doi:10.2215/CJN.07650810 21566106

[37] McGarity MZ, Herndon CN, Harris JA, Hobbs BF. Impact of satellite clinics on geographic access to assisted reproductive technology services in the United States. BMC Health Serv Res. 2022;22(1):928. doi:10.1186/s12913-022-08281-y 35854307 PMC9295342

[38] Wood BR, Bell C, Carr J, . Washington state satellite HIV clinic program: a model for delivering highly effective decentralized care in under-resourced communities. AIDS Care. 2018;30(9):1120-1127. doi:10.1080/09540121.2018.1481194 29852744 PMC6334292

[39] Raphael D, Bryant T, Mikkonen J, Raphael A. Social determinants of health: the Canadian facts. Ontario Tech University Faculty of Health Sciences and Toronto. York University School of Health Policy and Management. 2020. Accessed May 1, 2024. http://www.thecanadianfacts.org/

[40] Wilkinson R, Marmot M. Social Determinants of Health: The Solid Facts. 2nd ed. World Health Organization; 2003.

[41] Petric J, Handshin S, Jonnada PK, Karunakaran M, Barreto SG. The influence of socioeconomic status on access to cancer care and survival in resectable pancreatic cancer: a systematic review and meta-analysis. ANZ J Surg. 2022;92(11):2795-2807. doi:10.1111/ans.17964 35938456

[42] Munir MM, Endo Y, Alaimo L, . Impact of community privilege on access to care among patients following complex cancer surgery. Ann Surg. 2023;278(6):e1250-e1258. doi:10.1097/SLA.0000000000005979 37436887

[43] Schoen C, Osborn R, Squires D, Doty MM. Access, affordability, and insurance complexity are often worse in the United States compared to ten other countries. Health Aff (Millwood). 2013;32(12):2205-2215. doi:10.1377/hlthaff.2013.0879 24226092

[44] Chaudry A, Wimer C. Poverty is not just an indicator: the relationship between income, poverty, and child well-being. Acad Pediatr. 2016;16(3)(suppl):S23-S29. doi:10.1016/j.acap.2015.12.010 27044698

[45] Hahn RA, Barnett WS. Early childhood education: health, equity, and economics. Annu Rev Public Health. 2023;44:75-92. doi:10.1146/annurev-publhealth-071321-032337 36332658

[46] Ferguson H, Bovaird S, Mueller M. The impact of poverty on educational outcomes for children. Paediatr Child Health. 2007;12(8):701-706. doi:10.1093/pch/12.8.701 19030450 PMC2528798

[47] Cooper K, Stewart K. Does household income affect children’s outcomes? a systematic review of the evidence. Child Indic Res. 2021;14(3):981-1005. doi:10.1007/s12187-020-09782-0

[48] World Health Organization. Social determinants of health: key concepts. May 7, 2013. Accessed May 1, 2024. https://www.who.int/news-room/questions-and-answers/item/social-determinants-of-health-key-concepts

[49] Canadian Public Health Association. What are the social determinants of health? Accessed May 1, 2024. https://www.cpha.ca/what-are-social-determinants-health#f3

