# Supplementary material for: Sociodemographic Factors and Utilization of Pediatric Oncology Satellite Clinics in Ontario, Canada
Source: JAMA Netw Open. 2024 Dec 26;7(12):e2452063. doi: 10.1001/jamanetworkopen.2024.52063 (PMC11672161; doi:10.1001/jamanetworkopen.2024.52063)
Supplement: Supplement 1. — eTable. International Classification of Childhood Cancer (ICCC) Diagnostic groups eFigure 1. The geographic catchment areas around the Pediatric Oncology Group of Ontario’s eight satellite clinics eFigure 2. Sensitivity Analysis - Odds ratios for visiting a satellite clinic within one year of diagnosis, adjusted for geographic (distance-saved), socio-demographic and clinical factors, among children and youth diagnosed with a first, primary cancer between April 1, 2015, and March 31, 2022, in Ontario (n=1,280) eFigure 3. Sensitivity Analysis - Hazard ratios for visiting a satellite clinic after starting systemic therapy. adjusted for geographic (distance-saved), socio-demographic and clinical factors, among children and youth diagnosed with a first, primary cancer between April 1, 2015, and March 31, 2022, in Ontario (n=1,253) [file jamanetwopen-e2452063-s001.pdf]

Supplemental Online Content

Chiu M, Ali A, Leung F, et al. Sociodemographic factors and utilization of pediatric oncology satellite clinics in Ontario, Canada. *JAMA Netw Open*. 2024;7(12):e2452063. doi:10.1001/jamanetworkopen.2024.52063

eTable. International Classification of Childhood Cancer (ICCC) Diagnostic groups

eFigure 1. The geographic catchment areas around the Pediatric Oncology Group of Ontario's eight satellite clinics

eFigure 2. Sensitivity Analysis - Odds ratios for visiting a satellite clinic within one year of diagnosis, adjusted for geographic (distance-saved), socio-demographic and clinical factors, among children and youth diagnosed with a first, primary cancer between April 1, 2015, and March 31, 2022, in Ontario (n=1,280)

eFigure 3. Sensitivity Analysis - Hazard ratios for visiting a satellite clinic after starting systemic therapy. adjusted for geographic (distance-saved), socio-demographic and clinical factors, among children and youth diagnosed with a first, primary cancer between April 1, 2015, and March 31, 2022, in Ontario (n=1,253)

This supplemental material has been provided by the authors to give readers additional information about their work.

© 2024 Chiu M et al. *JAMA Network Open.*

eTable. International Classification of Childhood Cancer (ICCC) Diagnostic groups

**Leukemia and lymphoma**	**I.**	**Leukemias, myeloproliferative diseases and myelodysplastic diseases**
	a.	Lymphoid leukemias
	c.	Chronic myeloproliferative diseases
	d.	Myelodysplastic syndrome and other myeloproliferative diseases
	e.	Unspecified and other specified leukemias
	**II.**	**Lymphomas and reticuloendothelial neoplasms**
	a.	Hodgkin lymphomas
	b.	Non-Hodgkin lymphomas (except Burkitt lymphoma)
	c.	Burkitt lymphoma
	d.	Miscellaneous lymphoreticular neoplasms
	e.	Unspecified lymphomas
**CNS and germ cell CNS tumours**	**III.**	**CNS and miscellaneous intracranial and intraspinal neoplasms**
	a.	Ependymomas and choroid plexus tumour
	b.	Astrocytomas
	c.	Intracranial and intraspinal embryonal tumours
	d.	Other gliomas
	e.	Other specified intracranial and intraspinal neoplasms
	f.	Unspecified intracranial and intraspinal neoplasms
	**X.**	**Germ cell tumours, trophoblastic tumours and neoplasms of gonads**
	a.	Intracranial and intraspinal germ cell tumours
**Solid (non-CNS) tumours**	**IV.**	**Neuroblastoma and other peripheral nervous cell tumours**
	a.	Neuroblastoma and ganglioneuroblastoma
	b.	Other peripheral nervous cell tumours
	**V.**	**Retinoblastoma**
	**VI.**	**Renal tumours**
	a.	Nephroblastoma and other nonepithelial renal tumours
	b.	Renal carcinomas
	c.	Unspecified malignant renal tumours
	**VII.**	**Hepatic tumours**
	a.	Hepatoblastoma
	b.	Hepatic carcinomas
	c.	Unspecified malignant hepatic tumours
	**VIII.**	**Malignant bone tumours**
	a.	Osteosarcomas
	b.	Chondrosarcomas

© 2024 Chiu M et al. *JAMA Network Open.*

	c.	Ewing tumour and related sarcomas of bone
	d.	Other specified malignant bone tumours
	e.	Unspecified malignant bone tumours
	**IX.**	**Soft tissue and other extraosseous sarcomas**
	a.	Rhabdomyosarcomas
	b.	Fibrosarcomas, peripheral nerve sheath tumours and other fibrous neoplasms
	c.	Kaposi sarcoma
	d.	Other specified soft tissue sarcomas
	e.	Unspecified soft tissue sarcomas
	**X.**	**Germ cell tumours, trophoblastic tumours and neoplasms of gonads**
	b.	Malignant extracranial and extragonadal germ cell tumours
	c.	Malignant gonadal germ cell tumours
	d.	Gonadal carcinomas
	**XI.**	**Other malignant epithelial neoplasms and malignant melanomas**
	a.	Adrenocortical carcinomas
	b.	Thyroid carcinomas
	c.	Nasopharyngeal carcinomas
	d.	Malignant melanomas
	e.	Skin carcinomas
	f.	Other and unspecified carcinomas
	**XII.**	**Other and unspecified malignant neoplasms**
	a.	Other specified malignant tumours
	b.	Other unspecified malignant tumours

© 2024 Chiu M et al. *JAMA Network Open.*

eFigure 1. The geographic catchment areas around the Pediatric Oncology Group of Ontario's eight satellite clinics.

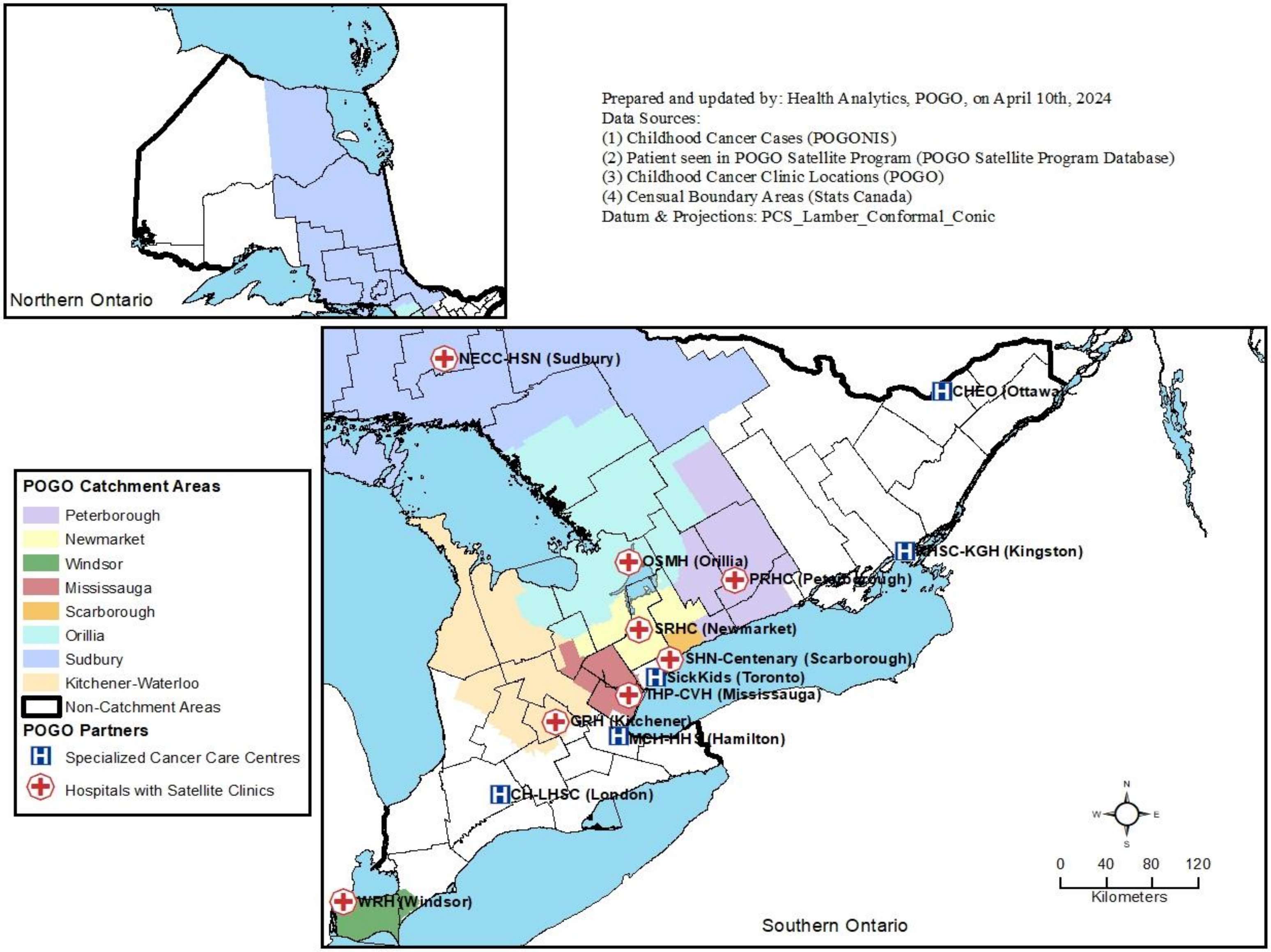

© 2024 Chiu M et al. *JAMA Network Open.*

eFigure 2. Sensitivity Analysis - Odds ratios for visiting a satellite clinic within one year of diagnosis, adjusted for geographic (distance-saved), socio-demographic and clinical factors, among children and youth diagnosed with a first, primary cancer between April 1, 2015, and March 31, 2022, in Ontario (n=1,280). Odds ratio on graph are presented on a logarithmic scale.

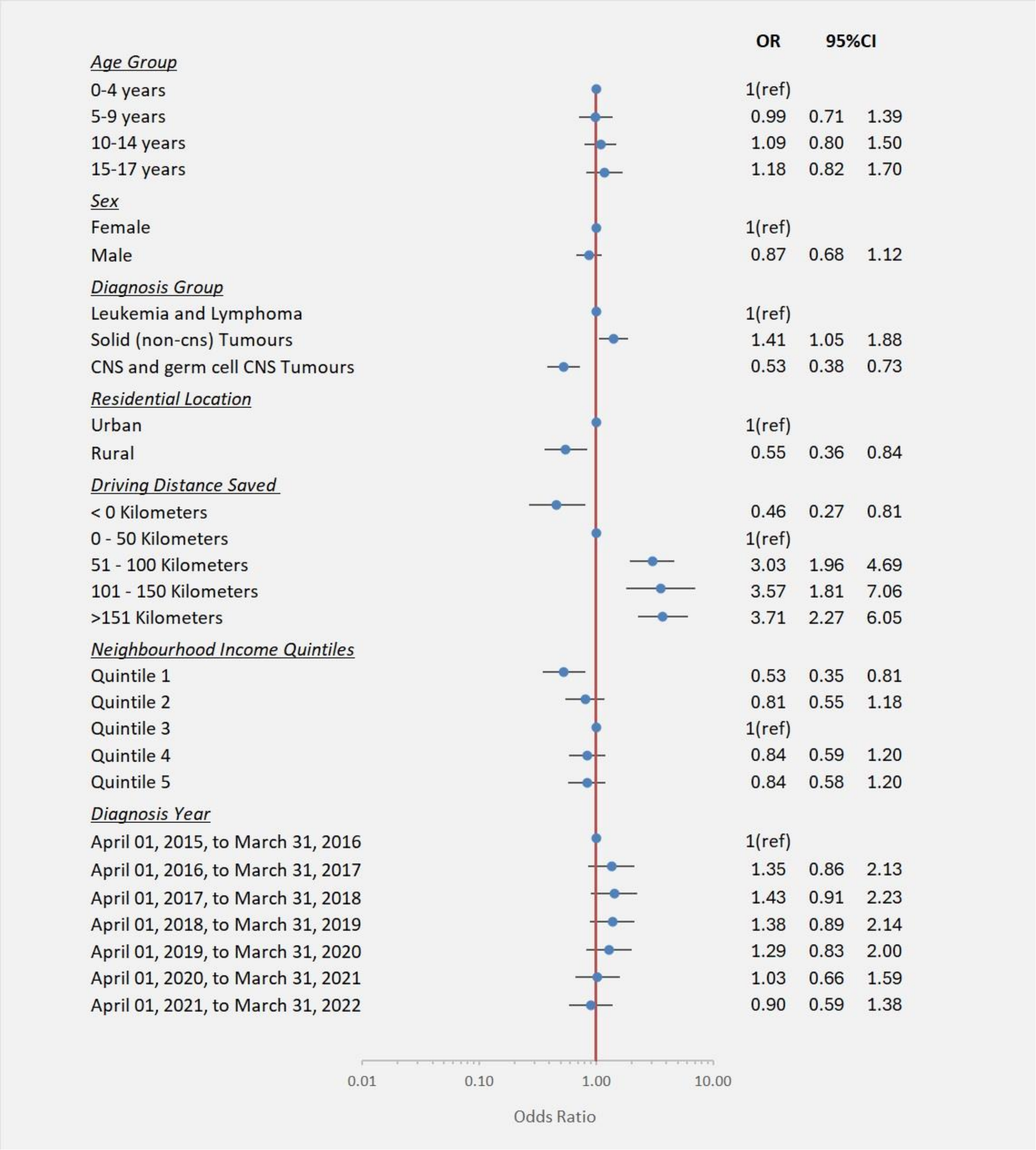

Driving Distance Saved = Shortest driving distance to satellite clinic – Shortest driving distance to specialized pediatric cancer centre. Patients whose driving distance to a specialized pediatric cancer centre is shorter than their driving distance to a satellite clinic will have driving distance saved values <0 km). Neighbourhood income quintile: 1(lowest) and 5(highest).

© 2024 Chiu M et al. *JAMA Network Open.*

eFigure 3. Sensitivity Analysis - Hazard ratios for visiting a satellite clinic after starting systemic therapy, adjusted for geographic (distance-saved), socio-demographic and clinical factors, among children and youth diagnosed with a first, primary cancer between April 1, 2015, and March 31, 2022, in Ontario (n=1,253). Hazards ratios on the graph are presented on a logarithmic scale.

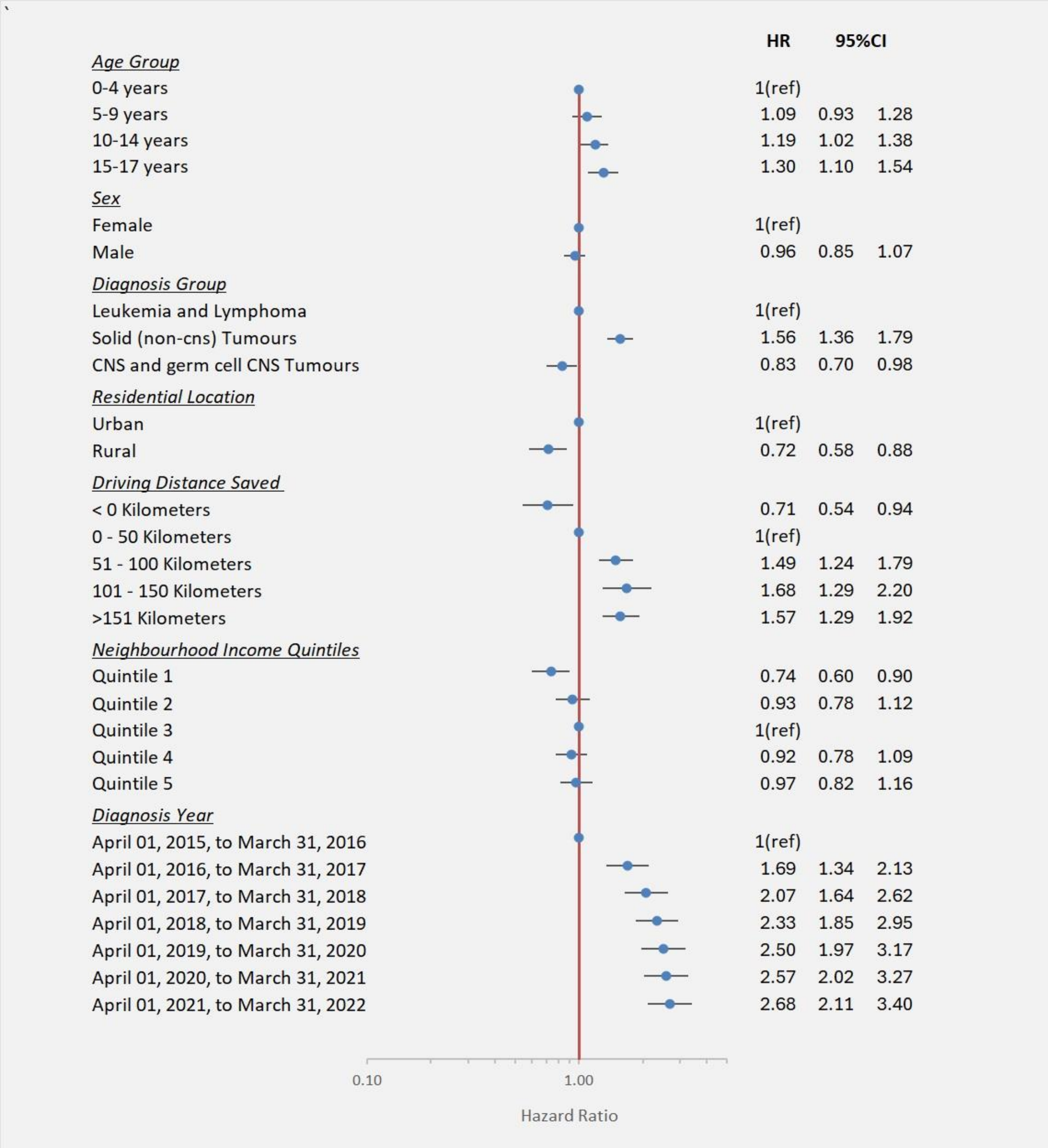

Driving Distance Saved = Shortest driving distance to satellite clinic – Shortest driving distance to specialized pediatric cancer centre. Patients whose driving distance to a specialized pediatric cancer centre is shorter than their driving distance to a satellite clinic will have driving distance saved values <0 km). Neighbourhood income quintile: 1(lowest) and 5(highest).

© 2024 Chiu M et al. *JAMA Network Open.*
