# Supplementary material for: Sociodemographic Factors and Utilization of Pediatric Oncology Satellite Clinics in Ontario, Canada
Source: JAMA Netw Open. 2024 Dec 26;7(12):e2452063. doi: 10.1001/jamanetworkopen.2024.52063 (PMC11672161; doi:10.1001/jamanetworkopen.2024.52063)
Supplement: Supplement 2. — Data Sharing Statement [file jamanetwopen-e2452063-s002.pdf]

Data Sharing Statement

Chiu. Sociodemographic Factors and Utilization of Pediatric Oncology Satellite Clinics in Ontario, Canada. *JAMA Netw Open*. Published December 26, 2024.
doi:10.1001/jamanetworkopen.2024.52063

Data
Data available: No

Additional Information
Explanation for why data not available: The dataset for this study is held securely at the Pediatric Oncology Group of Ontario (POGO). The data dictionary and underlying analytic codes are available from the authors upon request. Due to legal and privacy obligations, POGO is not permitted to make the dataset publicly available. POGO provides a formal data request process through its website (www.pogo.ca).
